# Osteoblastic flare in a patient with advanced gastric cancer after treatment with pemetrexed and oxaliplatin: implications for response assessment with RECIST criteria

**DOI:** 10.1186/1471-2407-7-94

**Published:** 2007-06-01

**Authors:** Vito Amoroso, Frida Pittiani, Salvatore Grisanti, Francesca Valcamonico, Edda Simoncini, Vittorio D Ferrari, Giovanni Marini

**Affiliations:** 1 Medical Oncology Unit, Beretta Foundation, Azienda Spedali Civili, Brescia, Italy; 2 Department of Radiology, University of Brescia, Brescia, Italy

## Abstract

**Background:**

The RECIST guidelines are commonly used in phase II and III clinical trials. The correct definition of response can be controversial in some situations, as in the case we describe.

**Case presentation:**

A 43 year-old man with advanced gastric cancer was enrolled in a phase II trial where he was treated with pemetrexed 500 mg/m^2 ^plus oxaliplatin 120 mg/m^2 ^every 3 weeks. At baseline, the target lesions were lymph-nodes, and the non-target lesions were small pulmonary nodules. At first re-evaluation, the target lesions showed partial response and the non-target lesions showed complete response, but new diffuse osteoblastic lesions appeared. The investigator decided to continue treatment until the second re-evaluation. CT scan confirmed the response of the target and non-target lesions, while the osteoblastic lesions did not change.

**Conclusion:**

The appearance of osteoblastic lesions after an active antitumor treatment, a phenomenon known as flare, can complicate the definition of the best overall response using RECIST criteria. This possibility should be considered by oncologists involved in clinical trials.

## Background

Phase II clinical studies that explore the activity of new drugs or new drug combinations demand a rigorous evaluation of tumor response. In the oncology community, the most commonly used criteria for response evaluation are contained in the RECIST guidelines [1]. We will describe here the case of a patient with advanced gastric cancer who was enrolled in a phase II trial. The evaluation of the best overall response for this patient has been problematic using the RECIST criteria, because these criteria do not contemplate a phenomenon documented in literature and well known in daily clinical practice called osteblastic flare.

## Case presentation

A 43-year-old man with a history of moderate hypertension and hypercholesterolemia consulted his physician for non-specific retrosternal pain. The first diagnostic tests ruled out myocardial ischemia. Due to the persistence of the symptoms, the patient underwent endoscopy of the upper gastrointestinal tract which revealed the presence of gastric adenocarcinoma in the antrum and corpus. The patient underwent a laparotomy. However, the direct infiltration of the tumor into the pancreatic head and the presence of small metastatic nodules in the omentum did not allow for a surgical resection.

**Figure 1:**
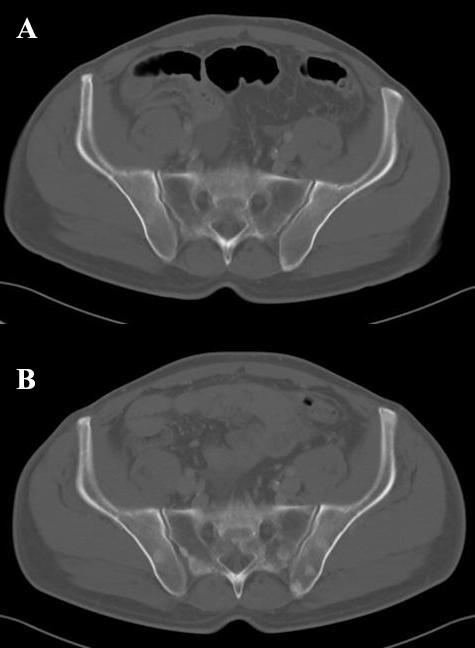
**A representative bone window scan of the pelvis**. In A, the bone window scan of the pelvis at baseline, before the administration of chemotherapy is shown. No clear osseous metastasis are seen. In B, the CT scan of the same region after two cycles of pemetrexed and oxaliplatin shows diffuse osteoblastic lesions which are apparent in the sacrum and iliac bones.

At the first examination by the medical oncologist the patient complained of moderate pain in the epigastric region with dorsal irradiation and dyspepsia. Codeine and acetaminophen were prescribed for symptom control. To complete tumor staging, a contrast-enhanced spiral computed tomography (CT) of the chest, abdomen, and pelvis was carried out which showed the presence of three pathological lymph-nodes. The lymph nodes had diameters larger than 10 mm and were located in the lesser curvature of the stomach, in the peripancreatic region, and in the mediastinum. Three pulmonary nodules that were smaller than 10 mm were also observed. A very small amount of ascites was present in the abdomen, but neither hepatic nor lytic bone lesions were detected. A representative bone window scan of the pelvis is shown in Figure 1A. The results of hematological and biochemical tests were normal with the exception of serum alkaline phosphatase, which was 2.5 times the upper limit of normal.

After a frank discussion of the benefits and the morbidity of chemotherapy, the patient agreed to enroll in a controlled phase II trial and was treated with pemetrexed 500 mg/m^2 ^and oxaliplatin 120 mg/m^2^, both repeated every 3 weeks. The first tumor re-evaluation was carried out 6 weeks after the beginning of the treatment, before the administration of the third cycle of chemotherapy. The three lymph-nodes, which were defined as target lesions, showed a 43% decrease in the sum of the longest diameters, classifying the response as partial. The pulmonary nodules, which were defined as non-target lesions, were no longer visible. The bone-window scans, however, revealed osteoblastic metastases in almost all vertebral bodies and in the pelvis, as shown in Figure 1B.

From the clinical point of view, the treatment was beneficial to the patient because it led to a significant reduction in the use of analgesics; moreover, the CA 19.9 level decreased from 222 U/ml to 66 U/ml.

We reasoned that the observed bone lesions were due to the strong osteoblastic response of preexisting bone metastases not visible on the baseline CT scan. In agreement with the patient, treatment was continued for two additional cycles. At the second tumor re-evaluation 6 weeks later, the target lesions were in confirmed partial response, and the bone metastases remained stable and no new blastic lesions appeared.

After the fifth chemotherapy cycle, the patient decided to go off the protocol because of intolerable side effects, particularly a pharyngeal disesthesia induced by oxaliplatin.

Two months later, the first follow-up CT showed new lymph-nodes in the mediastinum and in the mesentery, while the patient's performance status had deteriorated due to persistent vomiting. He was then referred to palliative care.

## Discussion

The RECIST guidelines to evaluate response to treatment in solid tumors are accurate, largely validated, and therefore used extensively in clinical trials [1]. However, these criteria do not take into account the osteosclerotic changes of bone metastases in response to active antitumor treatment [2,3]. We have described a patient with advanced gastric cancer who developed osteoblastic lesions in spite of a partial response after two cycles of chemotherapy with a regimen of pemetrexed and oxaliplatin.

Bone metastases occur in 1% to 11% of patients with gastric cancer; there is no apparent correlation with the histological tumor type, and the bone sites most frequently affected are the lumbar and thoracic vertebrae [4].

Chemotherapy can prolong overall survival and improve quality of life in patients with advanced gastric cancer, but the long-term results are not satisfactory [5]. It is therefore important to encourage participation in clinical trials evaluating new drugs.

The main goal of a phase II trial is to evaluate the objective response rate of a specific tumor type to an experimental treatment. According to the RECIST criteria, tumoral lesions are classified as measurable or non-measurable. Lesions with a diameter of at least 10 mm, precisely measurable in at least one dimension with spiral CT, belong to the first group. Non-measurable lesions, on the contrary, have a diameter less than 10 mm. Lesions which are truly non-measurable, such as abdominal-pelvic masses, lymphangitis, pleural or peritoneal effusion, and bone metastases also belong to this group [1].

Depending on the prevailing osteoclastic versus osteoblastic cellular activity, imaging techniques can identify bone metastases as lytic, blastic, or mixed. Distinct aspects of bone tissue can be visualized with different imaging approaches. Conventional radiography and CT visualize bone structure. Tumor and bone marrow can be visualized with magnetic resonance imaging. Osteoblastic bone metabolism can be visualized with skeletal scintigraphy, and tumor metabolism by positron emission tomography [6]. The existing standardized criteria from UICC and WHO for response assessment of bone metastases are based exclusively on conventional radiology and skeletal scintigraphy.

In the clinical case described here, the symptoms reported at initial presentation did not point to bone metastases. Furthermore, the standard examination protocols for asymptomatic cases like this one do not envisage skeletal scintigraphy. Therefore, the only indication of possible diffusion of the disease to bone was increased serum alkaline phosphatase.

Tumor re-evaluation after two cycles of chemotherapy indicated a partial response in the lymph-nodes identified as target lesions, and a complete response in the small pulmonary nodules identified as non-target lesions. Nevertheless, according to strict application of the RECIST criteria, the overall response could have been disease progression because of the appearance of new osteosclerotic lesions.

For decades, effective antitumor treatments have been known to increase the bone density of osteolytic metastases. Therefore, the appearance of new osteoblastic lesions is always difficult to interpret [7,8]. Skeletal scintigraphy is useful in some cases, but does not allow for a quantification of treatment response, and its application is limited by a phenomenon known as scintigraphic flare [9].

The RECIST guidelines do not take osteoblastic changes into account when treatment response is assessed. However, this possibility should be carefully considered, because an incorrect definition of disease progression can lead to the untimely interruption of a potentially beneficial treatment.

Outside the context of a clinical trial, oncologists usually weigh the treatment response of bone metastases against the beneficial effects of treatment on symptoms, and on the decrease of tumor markers. The decision whether to continue treatment depends mostly on the oncologist's experience rather than on published criteria [6].

In this clinical case the flare phenomenon was independent on the experimental treatment, pemetrexed and oxaliplatin. Therefore the occurrence of an osteoblastic response should be taken into consideration even in case of patients treated with standard cytotoxic regimens for gastric cancer, such as epirubicin, cisplatin and fluorouracil (ECF).

The patient described here received an effective treatment for two additional months before discontinuing due to cumulative toxic effects. Unfortunately, as is usually the case for advanced gastric cancer, the duration of response was very short. However, in patients with breast or prostate cancer the correct identification of an osteoblastic healing response to systemic treatments could lead to long-term clinical benefit [9].

## Conclusion

The RECIST criteria are frequently used to assess response in clinical trials investigating new treatments for solid tumors. However, the RECIST criteria are imperfect in precisely measuring tumor response and progression, and fall short in some situations and with some types of cancer therapeutics [10]. For this reason, it is important to report controversial cases that physicians often face in the conduct of phase II clinical studies. We believe it is reasonable for a phenomenon such as osteoblastic flare to be considered in the next version of RECIST guidelines.

## Competing interests

The author(s) declare that they have no competing interests.

## Authors' contributions

VA conceived the study, reviewed the literature and drafted the manuscript

FP was responsible for the evaluation of radiological images

SG was involved in the care of the patient and helped in manuscript preparation

FV was involved in the care of the patient and helped in manuscript preparation

ES participated in the conception of the study and reviewed the manuscript

VDF reviewed the literature and helped in editing the figures

GM provided expert guidance throughout the preparation of the manuscript, and reviewed the manuscript

All authors read and approved the manuscript

## Pre-publication history

The pre-publication history for this paper can be accessed here:



## References

[B1] Therasse P, Arbuck SG, Eisenhauer EA, Wanders J, Kaplan RS, Rubinstein L, Verweij J, Van Glabbeke M, van Oosterom AT, Christian MC, Gwyther SG (2000). New guidelines to evaluate the response to treatment in solid tumors. European Organization for Research and Treatment of Cancer, National Cancer Institute of the United States, National Cancer Institute of Canada. J Natl Cancer Inst.

[B2] Lokich JJ (1978). Osseous metastases: radiographic monitoring of therapeutic response. Oncology.

[B3] Pollen JJ, Shlaer WJ (1979). Osteoblastic response to successful treatment of metastatic cancer of the prostate. AJR Am J Roentgenol.

[B4] Nakanishi H, Araki N, Kuratsu S, Narahara H, Ishikawa O, Yoshikawa H (2004). Skeletal metastasis in patients with gastric cancer. Clin Orthop Relat Res.

[B5] Chau I, Norman AR, Cunningham D, Waters JS, Oates J, Ross PJ (2004). Multivariate prognostic factor analysis in locally advanced and metastatic esophago-gastric cancer–pooled analysis from three multicenter, randomized, controlled trials using individual patient data. J Clin Oncol.

[B6] Hamaoka T, Madewell JE, Podoloff DA, Hortobagyi GN, Ueno NT (2004). Bone imaging in metastatic breast cancer. J Clin Oncol.

[B7] Body JJ (1992). Metastatic bone disease: clinical and therapeutic aspects. Bone.

[B8] Vinholes J, Coleman R, Eastell R (1996). Effects of bone metastases on bone metabolism: Implications for diagnosis, imaging and assessment of response to cancer treatment. Cancer Treat Rev.

[B9] Vogel CL, Schoenfelder J, Shemano I, Hayes DF, Gams RA (1995). Worsening bone scan in the evaluation of antitumor response during hormonal therapy of breast cancer. J Clin Oncol.

[B10] Therasse P, Eisenhauer E, Verweij J (2006). RECIST revisited: a review of validation studies on tumour assessment. Eur J Cancer.

